# Abnormal Chromatin Folding in the Molecular Pathogenesis of Epilepsy and Autism Spectrum Disorder: a Meta-synthesis with Systematic Searching

**DOI:** 10.1007/s12035-022-03106-9

**Published:** 2022-11-11

**Authors:** Oliver Davis

**Affiliations:** Wellcome-MRC Cambridge Stem Cell Institute, University of Cambridge, Tennis Court Road, Cambridge, CB2 1QN UK

**Keywords:** Chromatin folding, Epilepsy, Autism, Pathogenesis

## Abstract

How DNA is folded and packaged in nucleosomes is an essential regulator of gene expression. Abnormal patterns of chromatin folding are implicated in a wide range of diseases and disorders, including epilepsy and autism spectrum disorder (ASD). These disorders are thought to have a shared pathogenesis involving an imbalance in the number of excitatory-inhibitory neurons formed during neurodevelopment; however, the underlying pathological mechanism behind this imbalance is poorly understood. Studies are increasingly implicating abnormal chromatin folding in neural stem cells as one of the candidate pathological mechanisms, but no review has yet attempted to summarise the knowledge in this field. This meta-synthesis is a systematic search of all the articles on epilepsy, ASD, and chromatin folding. Its two main objectives were to determine to what extent abnormal chromatin folding is implicated in the pathogenesis of epilepsy and ASD, and secondly how abnormal chromatin folding leads to pathological disease processes. This search produced 22 relevant articles, which together strongly implicate abnormal chromatin folding in the pathogenesis of epilepsy and ASD. A range of mutations and chromosomal structural abnormalities lead to this effect, including single nucleotide polymorphisms, copy number variants, translocations and mutations in chromatin modifying. However, knowledge is much more limited into how abnormal chromatin organisation subsequently causes pathological disease processes, not yet showing, for example, whether it leads to abnormal excitation-inhibitory neuron imbalance in human brain organoids.

**Supplementary Information:**

The online version contains supplementary material available at 10.1007/s12035-022-03106-9.

## Introduction

Atypical neurodevelopment is increasingly being shown to play a role in the pathogenesis of neurodevelopmental and neurological disorders. Epilepsy and autism spectrum disorder (ASD) provide prototypical examples of this; studies suggest they have a shared pathological aetiology in the form of atypical specification of neural progenitor cells (NPC), which leads to an imbalance in the number of excitatory and inhibitory neurons to form in the adult cortex [1]. However, it is less clear what causes this atypical NPC specification. The large number of high risk-of-pathology genes for ASD and epilepsy with functions in chromatin organisation suggests this may be a key mechanism [2, 3].

**Fig. 1:**
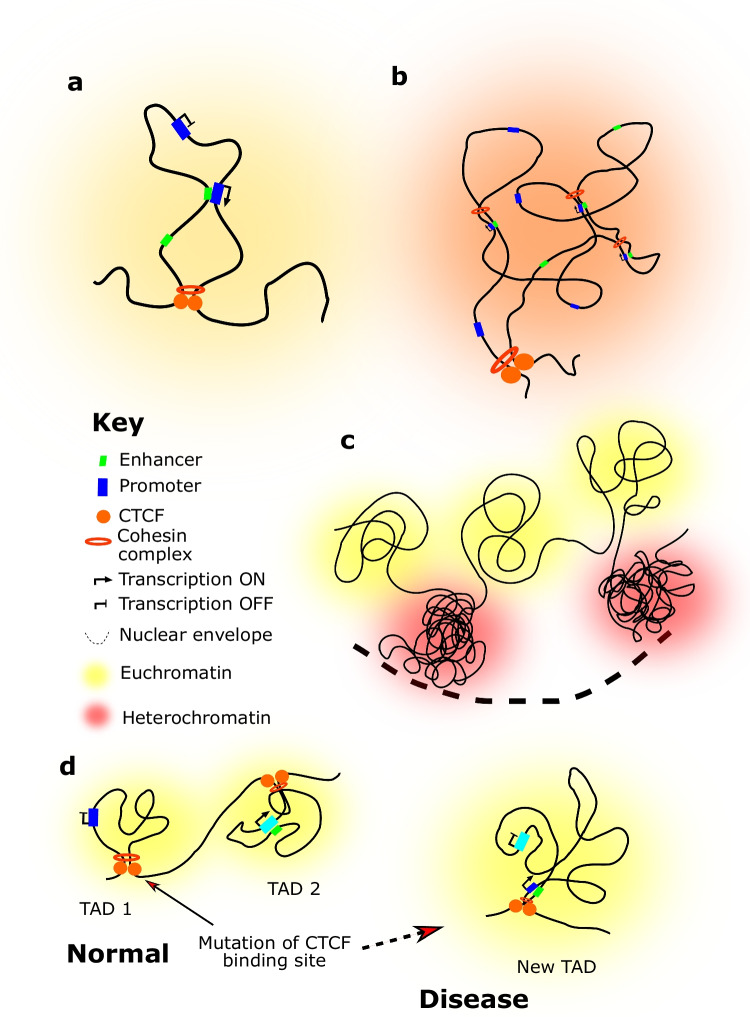
Chromosome topology at the level of chromatin loops (**a**), TADs (**b**) and A/B compartments (**c**). Deletion of CTCF binding sites can lead to formation of new TADs, which can cause genes to contact enhancers normally spatially too far away to do so (a process known as “enhancer hijacking”) (**d**). These new enhancer-promoter contacts can cause ectopic gene expression patterns that have pathological consequences

Chromatin folding is controlled at multiple scales (Fig. 1), each of which is implicated in controlling gene expression patterns and subsequent cell fate specification [4]. Usually at the smallest scale of several kilobases in length, but ranging up to several megabases, are chromatin loops [5]. These are stretches of DNA, anchored at the base by CTCF transcription factor and multiprotein cohesin and mediator complexes [6], that are brought together in close spatial proximity and exhibit high levels of contact probability (a measure of how frequently two DNA strands interact). This is important for bringing regulatory elements (like enhancers, which are stretches of DNA, often non-coding, that increase gene transcription) closer to their target gene promoters, which is an important transcriptional mechanism for controlling gene expression across development of an organism. At the larger scale of hundreds of kilobases are topologically associated domains (TAD). These are large stretches of DNA, often containing many chromatin loops, that show increased chromatin contact probability within their domains than with elsewhere in the genome. These organisational units, like chromatin loops, help to ensure that genes are spatially restricted from regulatory elements like enhancers and are another way to ensure gene expression is co-ordinately controlled across developmental time. On the scale of megabases are A and B compartments; A compartments consist of openly folded euchromatin and highly transcribed genes, whereas B compartments consist of heterochromatin, transcriptionally repressed genes and locations at the periphery of the nucleus. Chromatin in heterochromatic areas is folded much more tightly than in euchromatin, which restricts the access of transcriptional machinery (like RNA Polymerase II protein or transcription factors) to genetic material and prevents genes from being transcribed [6].

Abnormal patterns of chromatin topology are implicated in the pathogenesis of several diseases. Deletions, duplications and translocations can disrupt normal TAD architecture, causing fusions, shuffling, loss or new TADs to form. This subsequently re-organises regulatory elements into TADs they would not normally be within, which can cause abnormal enhancer-promoter interactions and subsequent ectopic gene expression patterns, a process known as “enhancer hijacking” [4]. Single nucleotide polymorphisms can target regulatory elements within a TAD, such as transcription factor binding sites or enhancer sequences. Mutations in transcription factors can also disturb chromosomal topology, such as for CTCF mutations that lead to abnormal chromatin interaction and accessibility patterns at enhancer sequences [7].

Abnormalities in chromosome topology are increasingly implicated in ASD and epilepsy; however, no review has yet attempted to synthesise the findings of the literature. Here, we present a meta-synthesis with systematic searching of studies on the role of abnormal chromatin topology in the aetiology of epilepsy and ASD. We seek to answer two primary questions:

- Question 1: Is abnormal chromatin topology implicated in the pathogenesis of epilepsy or ASD?
- Question 2: If implicated, how does abnormal chromatin topology contribute to epilepsy or ASD pathogenesis?

## Methods

Where relevant, this review has followed PRISMA guidelines whilst systematically searching for articles, which was performed using the following databases: Web of Science, Medline, Scopus and Embase. The same search criteria (below) were used for each database:

("chromatin loop*" OR "DNA loop*" OR "chromatin organisation" OR "chromatin folding" OR "enhancer promoter contact*" OR "enhancer-promoter contact*" OR "topologically associating domain*" OR "topologically associated domain*" OR "TAD" OR "chromatin domain*" OR "lamin associated domain*" OR "LAD" OR "euchromatin" OR "heterochromatin" OR "Hi-C" OR "chromosome confirmation capture") AND ("epilepsy" OR "epileptic" OR "seizure" OR "convulsion" OR "autism").

The results were screened by one reviewer using the following inclusion and exclusion criteria:

- Inclusion: primary biological studies (lab-based), peer-reviewed, specific techniques used in study [chromatin confirmation capture (CCC) OR assay for transposable accessibility sequencing (ATAC-seq) OR bulk/single cell RNA sequencing OR super-resolution microscopy], specific results [change in chromatin folding pattern, transcriptome OR genome folding]
- Exclusion: non peer-reviewed, reviews, non-English language, unpublished, no focus on chromatin folding (no CCC experiments/super res microscopy, ATAC-seq), not open access

**Fig. 2:**
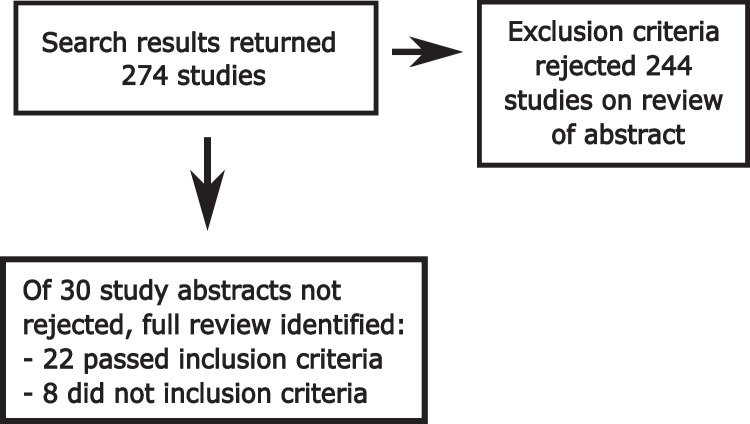
Flow diagram showing numbers of studies found and fully reviewed or excluded

The search identified 274 studies (see Fig. 2). Their abstracts were then reviewed using the inclusion and exclusion criteria. Two hundred forty-four studies were rejected based on initial inspection of the abstract and application of the exclusion and inclusion criteria, 30 were subsequently reviewed in full and 22 of these were subsequently determined to pass the inclusion criteria (these 22 articles are identified by an asterisk after the surname of the first author and identified in the Edexcel list of all articles found by the above search terms). As no consistent experimentation or outcome was present in each study, no consistent output metric could be extracted; a qualitative review and description of the results was instead conducted for a meta-synthesis. Studies were grouped according to shared mechanisms. All journal articles were accessed May 2022. Note this review has not been registered and a review protocol was not created.

In Table 1, example chromatin regulator genes implicated in the pathogenesis of epilepsy were identified from the EPI25 consortium [3] and for ASD from a large, recent case study (identified from the phenotypically categorise gene groups labelled as a chromatin modifier, or “mixed” and on further investigation identified to have epigenetic functions) [8].

**Table 1:** A list of chromatin regulators which, when mutated or carrying pathogenic variation, are implicated in the pathogenesis of ASC [8] or epilepsy [3]. Also included are non-chromatin regulator genes influenced by chromatin folding and discussed in this review

Gene	Function	ASD (A), epilepsy (E) or both (B)	References
ADNP*	Transcription factor with roles in controlling chromatin accessibility	A	[2, 9]
ANKRD11	Acetylates other proteins that control gene expression patterns	A	[8, 10]
ASH1L	Histone methyltransferase with a role in transcriptional activation	A	[8, 11]
AUTS2*	Transcriptional activator with important roles during neurodevelopment; roles in axon and dendrite guidance as well as neuronal migration during neurodevelopment	A	[2, 12]
CASK	X-linked serine protein kinase with roles in anchoring synaptic proteins and interacting with the transcription factor TBR1 to regulate gene expression patterns	B	[2, 3, 13]
CHD2	DNA helicase member of a chromatin remodeller complex	B	[2, 3, 14]
CHD8*	Part of a chromatin remodeller complex with roles regulating the transcription of other genes during development	A	[2, 15]
CNOT3	Binds to promoter sequences to act as a transcriptional repressor	A	[8, 16]
CTCF*	Transcription factor that can act as a transcriptional activator, repressor and insulator element	A	[2, 17]
DEAF1	Transcription factor that regulates the expression of other genes, partly by binding at promoter sites	A	[8, 18]
DNMT3A	Protein with a role in DNA methylation and control of gene expression patterns	A	[8, 19]
DRD2*	Encodes a dopamine receptor; role in regulating synaptic pruning and long-term depotentiation	A	[2, 20, 21]
FOXG1*	Pioneer transcription factor that acts as a transcription repressor during neurodevelopment, controlling cellular functions like proliferation	B	[2, 3, 22]
HDAC4	Histone deacetylase with roles in repressing transcriptional activity	B	[2, 3, 23]
HNRNPU	Binds DNA or RNA with diverse roles, including regulation of chromatin organisation in a transcription-dependant manner	B	[2, 3, 24]
KCTD7*	Encodes part of the potassium voltage-gated channel	E	[3, 25]
KDM5B	Histone demethylase that removes methylation marks at histone 3 lysine 4 and leads to transcriptional repression	A	[8, 26]
KDM6B*	Demethylates histones (particularly lysine 27 of histone 3), which has important roles during development	A	[2, 27]
KMT2A	Histone methyltransferase that deposits tri-methylation modification at histone 3 lysine 4	A	[8, 28]
KMT2C	Histone methyltransferase that deposits mono-methylation modification at histone 3 lysine 4	A	[8, 29]
LRFN5*	Cell adhesion molecular with roles in synapse formation	A	[2, 30]
MeCP2*	Transcription factor with roles in transcriptional repression during development	B	[2, 3, 31]
MED13L	Encodes a member of the Mediator complex and acts as a transcriptional co-activator	A	[8, 32]
MEF2C	Transcription factor involved in the development of various organ systems	B	[2, 3, 33]
NPTX2*	Structural protein involved in forming excitatory synapses	E	[34, 35]
PHF21A	Encodes a member of a histone deacetylase complex with roles in repression of gene expression	A	[8, 36]
POGZ*	Chromatin regulator with a role in controlling chromatin accessibility and cellular processes including mitosis	A	[2, 37]
RORB	Protein that binds hormone response elements upstream of target genes to enhancer their expression	B	[8, 38]
SLC6A1*	Encodes a GABA transporter	B	[2, 3, 39]
SMARCC2	Encodes a member of the SWI/SNF chromatin remodelling complex with roles in gene expression regulation	A	[8, 40]
SRCAP	Catalytic ATPase protein part of the SRCAP complex that remodels chromatin	A	[8, 41]
ZEB2	Binds to promoter sequences to act as a transcriptional repressor	E	[3, 42]

genes directly discussed in this article are indicated by an asterisk

## Results and Discussion

Mutations and mechanisms that disturb chromatin topology and may contribute to epilepsy or ASD pathogenesis can be simplified into two groups. The first concerns small-scale (single nucleotide polymorphisms) and large-scale (translocations and copy number variation) mutations of the structural elements themselves. The second concerns mutations of genes that encode proteins with roles in setting up or maintaining the architecture of chromatin topology, or its regulation, such as by epigenetic modifications and action potential cell–cell signalling.

### Mutations of Structural Elements

#### Single Nucleotide Polymorphisms

Common genetic variants in ASD, identified by genome-wide association studies (GWAS) of cases and controls, have been computationally studied in several papers. In one study, the SNP were mapped either directly to genes or, in those arising in non-coding DNA, their partner genes, which were identified based on chromatin interaction profiles and expression quantitative trait loci [43]. Mapped genes were enriched for expression in fetal tissue and gene ontology terms, including neuronal differentiation, GABAergic signalling and apoptosis. These results fit with the wider findings on the pathogenesis of ASD suggesting a neurodevelopmental basis involving an imbalance in the number of excitatory and inhibitory neurons [1]. It also provides evidence that non-coding DNA variants, which interact with genes, have a role in ASD pathogenesis.

Evidence of an association between ASD pathogenesis and perturbed enhancer-promoter interactions has been provided from studies of de novo mutations. In one study, de novo mutations were enriched at distal promoter sites, where transcription factors bind and interactions with enhancers are mediated [44]. More recent work has built on this by showing an enriched association with intronic regulatory elements (predicted by a machine algorithm on open chromatin regions) near ASD-associated genes [45]. The authors went a step further and showed reduced transcriptional activity of *SLC6A1* due to two de novo point mutations at an intronic enhancer in a neuroblastoma cell line. Interestingly, *SLC6A1* is a high-risk gene for both ASD and epilepsy, suggesting its dysregulation by perturbed enhancer function could contribute to epilepsy pathogenesis as well as ASD [2, 3]. These findings are supported by another study showing an enrichment of ASD-associated SNP in promoter-interacting regions and reduced expression of an ASD-associated gene (*DRD2*) upon monoallelic, CRISPR-mediated deletion of a downstream, intergenic, promoter-interacting region in iPSC-derived excitatory neurons [2, 20].

Having identified that enhancer dysfunction is a plausible candidate for understanding ASD pathogenesis, a question arises about whether and how commonly non-coding DNA mutations arise at these sites. However, computational analyses indicate they are not very common at these sites. In one study, only two ASD-associated SNP mapped (out of 86) to active enhancer regions present in cells of the adult brain [46]. Further evidence comes from Su et al., who made induced pluripotent stem cells from healthy volunteers and turned them into neural progenitor cells and cortical neurons. Throughout this process, cis-regulatory elements connected to gene promoters were identified, for which GWAS-identified ASD-associated SNP was overlapped and no enrichment was found [47]. Despite the findings of these studies, they are limited the number of ASD-associated SNP they can examine, which in turn is limited by the number of cases and controls that genetic studies can examine. As sample numbers increase, such as has already occurred in very recent publications [8], we will be better able to dissect the heritability of ASD, potentially identify other disease-linked SNP and more thoroughly investigate the role of small-scale mutations and polymorphisms at enhancer sequences as a pathogenic mechanism in ASD (and epilepsy).

Together, these studies show a clear association between ASD-associated single nucleotide polymorphisms and pathological enhancer-promoter interactions. Initial work has helped to provide a pathological link, but further research is needed to show the effect of these mutations and variations on the cellular context, such as whether dysregulated gene expression from perturbed enhancer-promoter dynamics leads to abnormal cell type specification. Further work is also needed to understand if there is a strong association between epilepsy-associated SNP and perturbed enhancer function.

#### Copy Number Variants (CNV)

Duplications and deletions of genomic areas can lead to changes in a gene copy number with local and widespread effects on gene expression and regulation. Several studies of syndromes in which ASD is common have shown this. For example, duplications and deletions of 16p11.2, a common cause of ASD, have been shown to cause widespread transcriptomic changes in lymphoblastoid cells [48]. These transcriptional changes were correlated with changes in chromosomal contact probability, suggesting that widespread, altered interactions with *cis* and *trans* regulatory elements are a consequence of CNV and a driver of the widespread transcriptomic change. Similar findings come from studies of people with duplication or deletion of the 16p11.2 BP2-BP3 interval, which is associated with an ASD diagnosis, and reduced or increased head circumference size [49].

Although evidence has shown CNV has widespread effects on genome folding, studies suggest the largest effects can occur in the local genomic area of the mutation. Research on 22q11 deletion, which causes DiGeorge syndrome (in which ASD is very common), show prominent changes in the regions flanking the deletion site, affecting CTCF binding patterns and topological domain organisation, as well as the pattern of histone modifications, including for H3K27 acetylation [50]. One consequence of this strongly altered chromatin topology in the area of the CNV is that distal genes with local regulatory elements become transcriptionally dysregulated. In a study of William-Beuren syndrome, in which ASD is very common, regions flanking the causative hemizygous deletion site (of an ~1.5 Mb region in 7q11.23) showed altered chromosomal interaction patterns and histone modifications, which correlated with changes in *AUTS2* expression, a high-risk gene for ASD [51]. Interestingly, the authors also observed a > 50% reduction in the chromosomal contacts between *KCTD7* gene, which is high risk for epilepsy [3], and a non-coding region containing possible regulatory elements. They suggest this shows chromatin looping can be allelically biased depending on allele-specific regulatory elements and expression, which fits with observations in other studies [52]. It also suggests allelic-based effects may be present in some of the other mutations discussed in this article, such as for SNPs at regulatory elements where homozygous carriers for variant alleles may have stronger phenotypes than heterozygous carriers.

Some of the effects described above, of local and widespread changes in chromosome topology, have been ascribed to the DNA regions targeted by CNV. A computational analysis by Fudenberg showed that structural deletions associated with ASD were enriched at strongly active transcriptional start sites and CTCF binding sites [53]. Deletions affecting these areas could be predicted to change TAD organisation, which is a mechanism by which dysregulated gene expression could be occurring [4]. Studies of patients with ASD or epilepsy, including Phelan-McDermid syndrome (in which ASD and recurrent seizures are common) and 22q11 deletion [50, 54], support this line of thinking; aetiological mutations arise at TAD boundaries and in some cases show an association with locally altered gene expression patterns [55].

In conclusion, these studies show a clear association between CNV and dysregulated gene expression through altered chromatin topology. However, these were conducted on tissues not pathologically implicated in ASD (lymphoblastoid cells) and do not provide mechanistic proof. Further work is required to show the associations in human neurological tissues, more strongly demonstrate an association for patients with epilepsy, and provide molecular proof of an aetiological mechanism.

#### Other Structural Mutations (Translocation and Sex-Specific Regulatory Patterns)

Translocations leading to changes in chromatin topology have been implicated in ASD and epilepsy pathogenesis. Their presence can cause a loss of normal regulatory elements, which data suggests is one cause of dysregulated *FOXG1* expression [56]. This gene is implicated as high risk for epilepsy [3], and abnormal expression during neurodevelopment can lead to FOXG1 syndrome in which ASD and epilepsy are especially common [57].

Reorganisation of local chromatin topology is another effect of translocations that can cause dysregulated gene expression. Balanced translocations of 5q14.3 in regulatory regions of the *MEF2C* gene are predicted to disrupt TAD boundaries and the activity of its enhancers. This is predicted to cause reduced expression of *MEF2C* in lymphoblastoid cells of patients with this aberration, as suggested by a change in chromosomal contact data [58]. Decreased *MEF2C* expression is caused in and a main driver of 5q14.3 microdeletion syndrome, in whom epilepsy and ASD-like features are characteristic. In another study of a patient with epilepsy and neurodevelopmental delay, a translocation of chromosomes 15 and 16 juxtaposed heterochromatic and euchromatic areas, which led to a change in the expression of the genes flanking the deletion sites [59]; the normally euchromatic genes became repressed whilst the heterochromatic genes became over-expressed. This final study emphasises that whilst translocations can perturb architectural elements of chromosomal topology, such as TAD boundaries, the local chromatin topology in the neighbouring chromosomal regions reciprocally influences the topology of the partner translocated chromosome region and their neighbouring genes.

Sex-specific regulation of chromosome topology is a recent mechanism implicated in ASD pathogenesis. A study of a family with high-functioning ASD males identified a maternally inherited topological haplotype at the *LRFN5* gene locus that was not present in the female siblings [60]. *LRFN5* is a high risk-of-pathology ASD gene that is located in the middle of a gene desert and large TAD [2]. It was further identified in this study that normal females and males have different histone modification patterns at this locus, further suggesting a sex-specific difference in chromosomal topology at this ASD-implicated gene [60].

The combined findings of these studies show abnormal chromatin topology due to translocations and sex-specific gene expression regulation are associated with ASD and epilepsy pathogenesis. However, no mechanistic investigation of these associations has been conducted and so the association have not yet been shown to be causal.

### Other Mechanisms Perturbing Chromatin Topology

The previous studies have focused on studying the impact of mutations and alterations of structural elements. However, other mechanisms have also been identified that alter chromatin folding and may contribute to the pathogenesis of epilepsy or ASD.

#### Mutations in Chromatin Regulators

Folding topology of the genome is strongly influenced and controlled by the actions of chromatin regulators. These regulators encompass a wide range of proteins, including transcription factors and chromatin regulators [61]. Studies implicate mutations in these chromatin regulators in the pathogenesis of ASD and epilepsy [3, 8, 62]. Moreover, based on the functional annotations of genes implicated as high risk, chromatin modification has been identified as a core mechanism to contribute to the pathogenesis of ASD [8]. Table 1 shows an example of the large number and wide function of these chromatin regulators, as well as the function of genes discussed in this article for which chromatin folding affects their expression.

Transcription factors have important roles in regulating chromatin topology, for example, through their effects on chromatin accessibility and enhancer activity [4]. In a study by Markenscoff-Papadimitriou et al., *POGZ*, a high-risk gene for ASD, showed important roles during mouse neurodevelopment in controlling gene expression patterns, including an enrichment for high-risk ASD genes [2, 37]. Their results suggest an aetiological mechanism whereby POGZ regulates the chromatin accessibility of transcriptional start sites and regulatory elements, including neuron-mapped enhancers. This is facilitated by its binding in partnership with low levels of ADNP, a chromatin remodeller, and HP1γ, a structural component of heterochromatin; genes downregulated in *POGZ* homozygous-deletion mutants showed low levels of ADNP and HP1γ binding in wild-type neurons (compared to upregulated or unchanged genes). Interestingly, *POGZ* expression-regulated target genes were also enriched for binding by the chromatin regulator *CHD8*, another high-risk ASD gene [2]. These results suggest mutations in chromatin regulators, mapped as high risk for ASD, pathologically converge on mis-regulated chromatin accessibility around regulatory elements (such as enhancers) and promoters (especially transcriptional start sites). They also suggest important roles for transcription factor dose (i.e. the amount of transcription factor binding), in this example ADNP levels, and combinatorial binding, in this example HP1γ and POGZ, in deriving normal patterns of chromatin accessibility in target genes.

Another way in which transcription factors control chromosome topology is by mediating the formation of liquid-like phase condensates, for example, in structures like heterochromatin. *MeCP2*, which when mutated causes Rett syndrome (in which epilepsy and ASD-like symptoms are prevalent), plays an essential role in this process [63]. In mouse neurons, patient-derived mutations disrupt the ability of heterochromatin to form condensates and lead to widespread decreased transcriptional activity in euchromatic genes [63]. This suggests that the normal chromosomal topology of large areas of the genome are disturbed in Rett syndrome. To verify this, it would be interesting to examine for changes in chromosome contact probability maps of the mouse neuronal cells bearing patient-derived *MeCP2* mutations.

#### Altered Epigenetic Modifications

Research has shown that altered epigenetic modifications occur in ASD over genomic areas important for the structural organisation of chromatin. In a post-mortem study of ASD patient brain tissue, it was shown that the DNA 5-hydroxymethylcytosine mark, which controls gene expression patterns, differs across the genome [64]. Most differentially hydroxymethylated areas of the genome were intergenic, with many occurring over TAD boundaries and up to 38.6% being associated with enhancers. Analysis of genes associated with these intergenic regions showed an enrichment for genes associated with ASD. Interestingly, studies suggest enhancer hydroxymethylation is important for transcription by maintaining an open chromatin state, providing access to proteins like CTCF, which are needed for TAD formation [65]. It would be interesting to see if this occurs at enhancers important for the expression of ASD high-risk genes and their local chromosomal topology.

Many genes mapped as high risk for epilepsy have broad enhancer-like domains (BELD), for which most of their gene body exhibits open chromatin and bears the H3K27 acetylation and H3K4 mono-methylation histone modifications [66]. These genes are downregulated in individuals with idiopathic ASD and are sensitive to altered expression of chromatin regulators mapped as high risk for ASD, including *CHD8* and *KDM6B* [2]. Interestingly, BELD genes show an increased chromatin interaction frequency across their gene body, rather than for non-BELD genes or promoter interactions with enhancers outside of the BELD. It would be interesting to test whether the histone modifications across the BELD regions facilitate their increased intra-gene chromosomal contact probability, and whether their loss impacts on synapse function, which BELD genes are functionally enriched for using gene ontology terms [66].

#### Action Potential-Based Regulation of Chromosome Topology

Recent data has shown that action potentials received by cortical neurons can rapidly alter the chromosome topology of rapidly transcribed genes as well as induce longer-lasting changes in chromatin loop formation [67]. In a study by Beagan et al., mouse cortical neurons exhibited pre-formed chromatin loops with anchoring enhancers whose H3K27 acetylation modification was deposited upon electrical stimulation. ASD-associated SNPs were enriched at these chromatin loops, which in wild-type mice connected to genes showing increased expression following electrical activity. It would be interesting to extend this work to ASD patient cohorts to see whether there are deficiencies in the pre-formation of these loops or alternatively of the ability of the anchored enhancers to alter gene transcription upon electrical activation.

Neuronal activation in a model of status epilepticus in mice has also shown that synaptic activity leads to acute and chronic topological changes in chromatin folding. In a study by Fernandez-Albert et al., changes were seen over hours and days in neuronal transcriptomes and chromatin accessibility at genes and regulatory elements (including enhancers) [34]. These changes correlated with chromosome confirmation capture experiments; within 1 h, the TSS of genes expressed following electrical stimulation showed a much increased interaction with nearby dynamically accessible regions; after 48 h, although the chromosome topology had largely returned to baseline, some promoter-enhancer contacts formed following electrical stimulation persisted and were associated with increased transcription, including for genes encoding synaptic proteins, such as *NPTX2*.

These results show that chromatin topology is altered following seizure-induced activity. It would be interesting to see how the changes in chromosome topology due to seizure activity influence the future patterns of electrical activity in affected brains. Similarly, it would be interesting to see how the chromosome topology of a brain with excitatory-inhibitory imbalance responds to electrical activity as in these experiments, as well as whether mutations in high-risk epilepsy genes alter the normal response in topological re-arrangement following seizure activity.

### Future Considerations

Beyond the focus of this review, the chromatin-based mechanisms discussed in this paper provide numerous therapeutic targets to investigate. This is especially relevant given current anti-epileptic drugs (AED) are known to have wide-spread epigenetic effects [68]. For example, valproic acid is one of the most commonly used AEDs [69]. Studies have shown it inhibits DNMT3a expression and is a non-selective inhibitor of histone deacetylases [70, 71]. However, the widespread targeting of epigenetic marks and expression of chromatin regulators by AED may also underly its known teratogenicity [72], which may also help to explain, for example, how VPA-treatment of rodents leads to an ASD-model [73]. More detailed studies on how AEDs affect chromatin folding in adult and neurodevelopmental neurons will help to uncover their underlying epigenetic mechanisms causing adverse drug effects. Similarly, further work is needed to investigate the use of more targeted drugs, which inhibit specific chromatin marks of chromatin regulators, on pathogenic models of ASD and epilepsy, such as brain organoids. A more targeted approach may circumvent the more challenging aspects of current AEDs with their adverse effects, especially in the context of development where the goal may be to circumvent the risk of developing ASD or epilepsy.

In addition to therapeutics, further consideration needs be given to environmental influences that are implicated in pathologies with a neurodevelopmental basis and have been shown to alter gene expression patterns [74]. For example, changes in DNA methylation have been shown at numerous genomic loci due to maternal smoking [75], poor dietary habits such as low folate intake [76], caesarean section delivery (compared to natural birth) [77] and parental behaviour [78]. These changes also extend to other epigenetic markers, such as histone modifications in relation to parental behaviour [78]. It would be interesting to study how chromatin folding changes in response to environmental stimuli such as those described above, which may help identify reversible and druggable molecular targets in at-risk individuals. It may also help to explain the way in which some of these environmental stimuli are associated with an increased risk of disorders like ASD [79].

## Conclusion

**Fig. 3:**
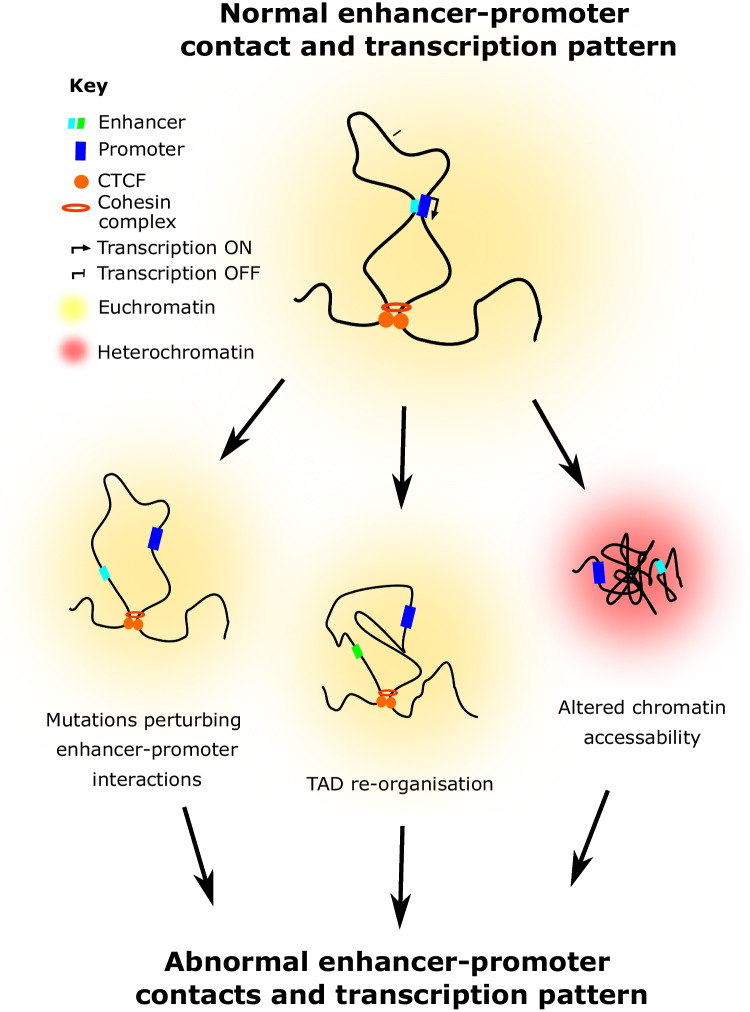
Abnormal chromatin folding perturbs normal enhancer-promoter contacts and subsequent transcriptional patterns. Mutations and genetic structural modifications converge on mechanisms perturbing normal enhancer-promoter contact formation, including TAD re-organisation, altered chromatin accessibility and mutations at the enhancer or promoter DNA sequences preventing their normal association. These general mechanisms then pathologically converge on abnormal transcriptional outputs (including excessive or no transcriptional activity)

This review provides strong evidence implicating abnormal chromatin folding in the pathogenesis of epilepsy and ASD. This can be due to small or large scale genetic alterations, or less direct mechanisms like mutations of transcription factors. These pathological processes converge on general mechanisms whose common output is to perturb normal enhancer-promoter contact formation and lead to abnormal gene expression patterns (Fig. 3).

Much less mechanistic research has been done to study the pathological consequences and causality of abnormal promoter-enhancer contacts. Initial work has shown that disease-associated SNPs at enhancers can reduce transcription at target genes, whilst other studies have identified that action potentials regulate epigenetic modifications at enhancers in pre-formed chromatin loops (sites also enriched in disease-associated SNPs). Much more work is required to go beyond these initial insights. For example, what pathological cellular processes arise from the abnormal enhancer-promoter contacts and the aberrant transcription patterns they cause? Answers to these and other questions, using patient-derived brain organoids, are needed to provide new insights into the pathogenesis of epilepsy and ASD. This, in turn, will lead to new insights into how to diagnose and treat these conditions.

## Supplementary Information

Below is the link to the electronic supplementary material.

Supplementary file1 (XLSX 61 KB)

Supplementary file2 (DOCX 34 KB)

## Data Availability

Not applicable (supplementary data submitted with article related to PRISMA guidelines and results of systematic searching).
